# Culture of Clinical Specimens Reveals Extensive Diversity of Legionella pneumophila Strains in Arizona

**DOI:** 10.1128/mSphere.00649-18

**Published:** 2019-02-27

**Authors:** Brian H. Raphael, Trung Huynh, Ellen Brown, Jessica C. Smith, Irene Ruberto, Linda Getsinger, Stacy White, Jonas M. Winchell

**Affiliations:** a Respiratory Diseases Branch, Centers for Disease Control and Prevention, Atlanta, Georgia, USA; b Arizona Department of Health Services, Phoenix, Arizona, USA; University of Nebraska Medical Center

**Keywords:** *Legionella pneumophila*, clinical microbiology, genomic epidemiology, public health, whole-genome MLST

## Abstract

Between 2000 and 2017, a total of 236 *Legionella* species isolates from Arizona were submitted to the CDC for reference testing. Most of these isolates were recovered from bronchoalveolar lavage specimens. Although the incidence of legionellosis in Arizona is less than the overall U.S. incidence, Arizona submits the largest number of isolates to the CDC for testing compared to those from other states. In addition to a higher proportion of culture confirmation of legionellosis cases in Arizona than in other states, all Legionellapneumophila isolates are forwarded to the CDC for confirmatory testing. Compared to that from other states, a higher proportion of isolates from Arizona were identified as belonging to L. pneumophila serogroups 6 (28.2%) and 8 (8.9%). Genome sequencing was conducted on 113 L. pneumophila clinical isolates not known to be associated with outbreaks in order to understand the genomic diversity of strains causing legionellosis in Arizona. Whole-genome multilocus sequence typing (wgMLST) revealed 17 clusters of isolates sharing at least 99% identical allele content. Only two of these clusters contained isolates from more than one individual with exposure at the same facility. Additionally, wgMLST analysis revealed a group of 31 isolates predominantly belonging to serogroup 6 and containing isolates from three separate clusters. Single nucleotide polymorphism (SNP) and pangenome analysis were used to further resolve genome sequences belonging to a subset of isolates. This study demonstrates that culture of clinical specimens for *Legionella* spp. reveals a highly diverse population of strains causing legionellosis in Arizona which could be underappreciated using other diagnostic approaches.

**IMPORTANCE** Culture of clinical specimens from patients with Legionnaires’ disease is rarely performed, restricting our understanding of the diversity and ecology of *Legionella*. Culture of *Legionella* from patient specimens in Arizona revealed a greater proportion of non-serogroup 1 Legionellapneumophila isolates than in other U.S. isolates examined. Disease caused by such isolates may go undetected using other diagnostic methods. Moreover, genome sequence analysis revealed that these isolates were genetically diverse, and understanding these populations may help in future environmental source attribution studies.

## Abstract (precis)

Culture of clinical specimens from patients with Legionnaires’ disease is rarely performed, restricting our understanding of the diversity and ecology of *Legionella*. Culture of *Legionella* from patient specimens in Arizona revealed a greater proportion of non-serogroup 1 Legionellapneumophila isolates than in other U.S. isolates examined. Disease caused by such isolates may go undetected using other diagnostic methods. Moreover, genome sequence analysis revealed that these isolates were genetically diverse, and understanding these populations may help in future environmental source attribution studies.

## INTRODUCTION

Legionnaires’ disease (LD) is a serious and potentially fatal bacterial pneumonia caused by various species of *Legionella*. Although *Legionella* can be found in natural water sources (e.g., lakes, rivers, and streams), the occurrence of LD in humans is due to the inhalation or aspiration of the organism present in potable and nonpotable human-made water systems. The incidence of LD in the United States has increased nearly 4-fold since 2000 (1, 2). Although the causes of this increase are not completely understood, many factors, including increased diagnostic testing, clinician awareness, expanding susceptible populations, and warmer temperatures, likely play a role.

Legionellosis is characterized by two distinct illnesses, LD and Pontiac fever. LD is associated with fever, cough, and pneumonia. In contrast, Pontiac fever is typically a self-limiting flu-like illness. A confirmed case of legionellosis requires clinically compatible symptoms and at least one confirmatory laboratory test, including isolation of *Legionella* from respiratory secretions (or other normally sterile sites), detection of Legionella pneumophila serogroup 1 (Lp1) using the urinary antigen test (UAT), and/or a 4-fold or greater rise in antibody titer to Lp1 antigen between acute and convalescent-phase serum (https://www.cdc.gov/legionella/health-depts/surv-reporting/case-definitions.html).

According to the National Notifiable Diseases Surveillance System (3), the incidence of legionellosis in Arizona for 2016 was 1.10 cases per 100,000 individuals. The overall U.S. incidence in 2016 was 1.9 cases per 100,000 individuals, and the state with the highest incidence was Ohio (4.33 cases per 100,000 individuals). In Arizona, only 3% of cases reported between 2010 and 2017 were considered health care associated; however, nearly 20% of cases nationally are considered health care associated (4). While the surveillance definition of a possible or definite health care-associated LD case in some public health jurisdictions involves any exposure to a health care facility during the incubation period, the definition in Arizona (since 2016) requires the patient to have an overnight stay in a health care facility.

Although the most common laboratory diagnostic assay for LD is the UAT which can detect Lp1, culture of lower respiratory tract specimens (such as sputum, bronchoalveolar lavage [BAL] fluid, etc.) permits the detection of any *Legionella* species or serogroup but requires extended incubation (up to 14 days), and the recovery of isolates can be inhibited by the administration of antibiotics prior to specimen collection. Moreover, culture of *Legionella* spp. requires specialized media such as buffered charcoal yeast extract (BCYE), which is often supplemented with various antibiotics to restrict growth of other contaminating microorganisms (5). More recently, nucleic acid-based tests such as PCR on respiratory specimens have shown sensitivity exceeding that of culture (6), but the lack of commercially available tests hinders the widespread use of these assays. Ideally, both a urine specimen and lower respiratory tract specimen should be collected simultaneously for LD testing. Of 2014 and 2015 U.S. LD cases reported to the CDC for which laboratory confirmation was available, approximately 98% were confirmed by UAT (4). Between 2006 and 2017, a total of 679 cases of LD were reported in Arizona, and 73.6% of these cases were confirmed by UAT. Remarkably, 25.2% of AZ cases were confirmed by culture, whereas only ∼4% of U.S. LD cases are culture confirmed.

Although Arizona is not among the states with the highest incidence of LD in the United States, the availability of a large number of clinical isolates provided a unique opportunity to characterize the population of L. pneumophila strains causing disease in Arizona. In this study, we compared the serogroups of L. pneumophila clinical isolates recovered in Arizona with those from other U.S. states and analyzed the genomic sequences of a subset of these isolates to determine the diversity of clinically significant L. pneumophila in this unique environment.

## RESULTS

### Summary of *Legionella* reference testing 2000 to 2017.

**FIG 1:**
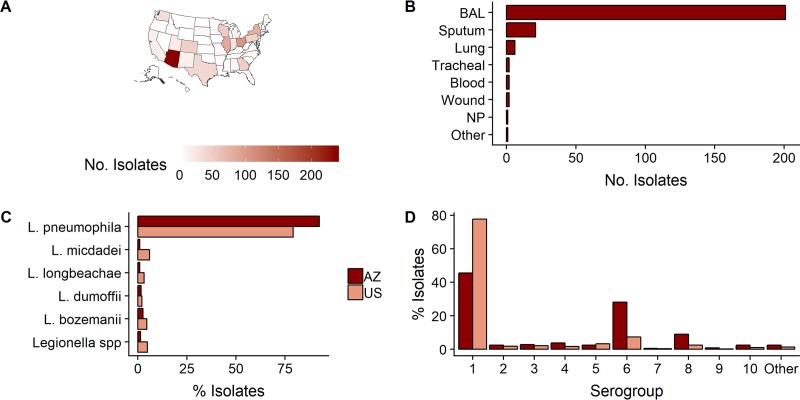
Properties of clinical isolates submitted to the CDC (2000 to 2017). (A) Numbers of clinical *Legionella* species isolates submitted by various states. Nearly 25% of isolates submitted to the CDC from 2000 to 2017 came from Arizona. (B) Numbers of *Legionella* species isolates recovered from various clinical specimens submitted by Arizona. BAL, bronchoalveolar lavage; NP, nasopharyngeal swab. (C) Proportions of various *Legionella* spp. submitted to the CDC by Arizona compared to all other states. (D) Proportions of different L. pneumophila serogroups submitted to the CDC by Arizona compared to all other states.

The CDC conducts reference testing on *Legionella* isolates submitted by U.S. public health laboratories; however, isolate submissions practices are not uniform. Nonetheless, 1,036 suspected *Legionella* isolates recovered from clinical sources were submitted to the CDC by 43 states between 2000 and 2017 (Fig. 1A). Arizona submitted 236 of these isolates. The states associated with the next three highest submissions (Ohio, Illinois, and New York) were in high-LD-incidence regions (4). Over 85% of the AZ isolates were recovered from BAL specimens, and only ∼9% of AZ isolates were recovered from sputum (Fig. 1B).

Arizona submitted a larger proportion of isolates (92.6%) that were confirmed as L. pneumophila than other U.S. states (79.2%) (Fig. 1C). The proportion of AZ Lp1 isolates (45.5%) was lower than the proportion (77.6%) submitted from other states (Fig. 1D). Conversely, the proportions of AZ Lp6 and Lp8 isolates (28.2% and 8.9%, respectively) were higher than those submitted by other states (8.9% and 2.5%, respectively).

### Genomic sequence diversity of AZ L. pneumophila clinical isolates.

A total of 113 L. pneumophila isolates recovered from a subset of LD patients in Arizona were sequenced (see Table S1 in the supplemental material). The isolates were recovered between 2008 and 2017. To characterize isolates associated with sporadic disease, those from known outbreaks were omitted. Lp1 (*n* = 47) and Lp6 (*n* = 30) accounted for more than two-thirds of the isolates sequenced. Interestingly, the sequence types (STs) extracted from the genome sequences were more diverse among the Lp1 isolates (21 STs) than among the Lp6 isolates (7 STs).

Whole-genome multilocus sequence typing (wgMLST) analysis revealed a large clade (clade A) of 31 isolates containing 24 Lp6 isolates which belonged to either ST68, ST187, or ST242. Conversely, two Lp1 isolates (AZ00035500 and AZ00022273) belonging to ST59 and ST115, respectively, were identified on two separate long branches in the wgMLST dendrogram and shared <10% allele similarity with any other isolate examined.

### Cluster analysis.

**FIG 2:**
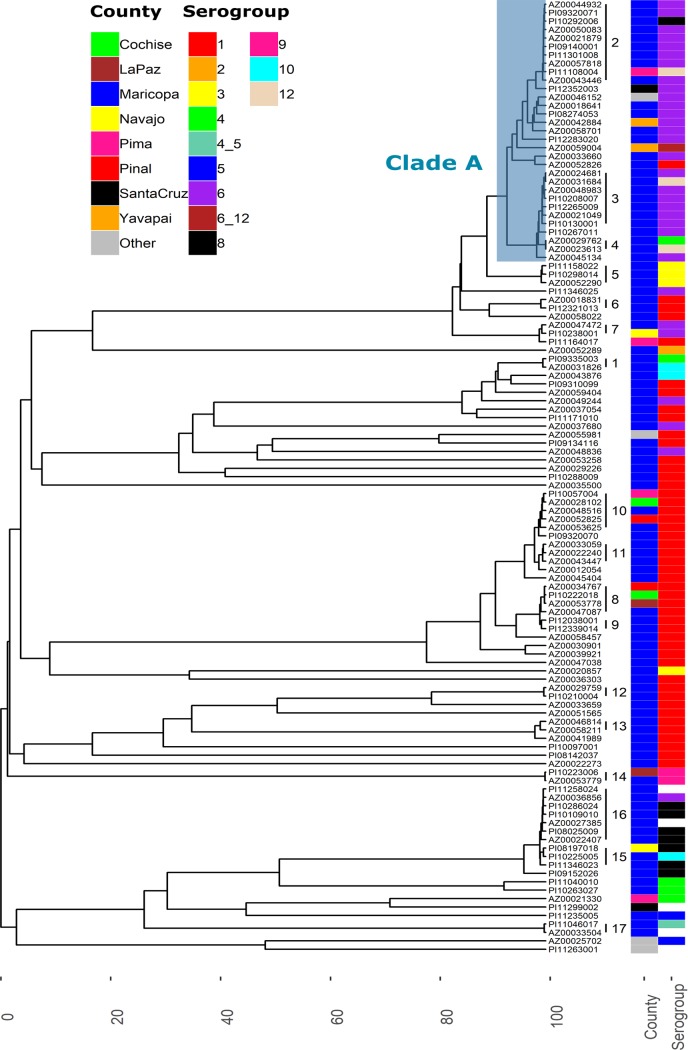
wgMLST analysis. The UPGMA dendrogram was built using a pairwise similarity matrix of wgMLST profiles. Clusters of isolates sharing at least 99% allele identity are numbered. The county of residence of the individuals from which the isolates were recovered and the isolate serogroups are shown in a corresponding matrix of colored blocks. The blue shaded block denotes clade A.

Using a threshold of ≥99% allele similarity, 17 clusters of 2 or more isolates were detected (Fig. 2; see also Table S1 in the supplemental material). Six of these clusters contained isolates belonging to more than one serogroup. Clusters 5, 8, and 15 involved isolates recovered from individuals with known similar exposures over multiple years. Cluster 5 was composed of three isolates recovered from bronchial washings conducted at the same hospital (hospital A) between 2010 and 2017. Remarkably, one of the individuals from which an isolate was recovered was asymptomatic. Cluster 8 involved 4 isolates recovered from individuals residing in 4 separate counties between 2010 and 2017. The clinical specimens from 3 of these individuals were collected at the same hospital (hospital B), and one of these cases may have been nosocomial. Notably, all of the isolates associated with cluster 15 were recovered from individuals whose respiratory specimens were collected at the same facility between 2008 and 2010, although none of the individuals had overnight exposures. Two additional clusters contained isolates from individuals with possible nosocomial infections; however, other isolates from the same clusters were recovered from patients who were treated in different facilities.

10.1128/mSphere.00649-18.1 TABLE S1 Properties of isolates sequenced in this study. Download Table S1, XLSX file, 0.1 MB. Copyright © 2019 Raphael et al. 2019 Raphael et al. This content is distributed under the terms of the Creative Commons Attribution 4.0 International license.

**FIG 3:**
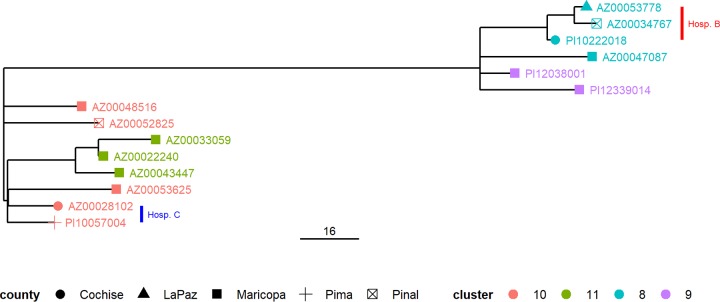
SNP analysis of ST1 isolate clusters. A phylogeny reconstruction based on SNP differences in regions outside those predicted to be due to recombination among isolates associated with clusters 8 through 11 is shown. The county of residence of the individuals from which the isolates were recovered are depicted by different shapes shown in the legend, and the tip labels are color coded according to the isolate cluster determined in the wgMLST analysis.

ST1 strains are highly conserved at the genetic level and represent the most common population of L. pneumophila (7). SNP analysis was conducted to resolve the ST1 isolates associated with clusters 8 through 11. After removing putative recombination sites, the isolates formed two distinct subclades composed of isolates from clusters 8 and 9 in one subclade and clusters 10 and 11 in the other subclade (Fig. 3). Interestingly, isolates from cluster 10 did not share an internal node, suggesting that the individuals from which these isolates where collected may not have shared a common source. Three of the four isolates associated with cluster 8 formed a subclade. Remarkably, this clade corresponded to isolates recovered from individuals whose clinical specimens were collected at hospital B. Similarly, two of the four isolates associated with cluster 10 where the original clinical specimens were collected at the same hospital (hospital C) formed a subclade.

In a previous study, a large (∼132-kb) plasmid (termed pLPP in L. pneumophila strain Paris) was variably present among outbreak-associated ST1 isolates (8). In this study, the genome assemblies of the ST1 isolates forming clusters 8 to 11 were also examined for nucleotide similarity to pLPP (see Fig. S1). Only one of two isolates in cluster 9 and one of five isolates in cluster 10 lacked significant similarity with pLPP. Notably, the two isolates recovered from clinical specimens collected at hospital C appear to harbor nucleotide similarity with pLPP.

10.1128/mSphere.00649-18.2 FIG S1 BLAST ring image of ST1 isolate clusters with pLPP. Isolate sequences associated with clusters 8 through 11 were compared to the L. pneumophila strain Paris plasmid (pLPP) using BLAST. Regions containing at least 70% nucleotide sequence identify are shaded. Rings are color coded according to the isolate cluster determined in the wgMLST analysis: cluster 8, red; cluster 9, orange; cluster 10, green; cluster 11, blue. The isolates depicted starting with the innermost ring are PI10222018, AZ00034767, AZ000047087, AZ00053778, PI2038001, PI12339014, PI10057004, AZ00028102, AZ00048516, AZ00052825, AZ00053625, AZ00022240, AZ00033059, and AZ00043447. Download FIG S1, PDF file, 0.3 MB. Copyright © 2019 Raphael et al. 2019 Raphael et al. This content is distributed under the terms of the Creative Commons Attribution 4.0 International license.

### Pangenome analysis.

Most of the isolates associated with clade A (Fig. 2) belonged to serogroup 6. Pangenome analysis was used to determine if these isolates contained additional gene content that could be used to improve resolution.

**FIG 4:**
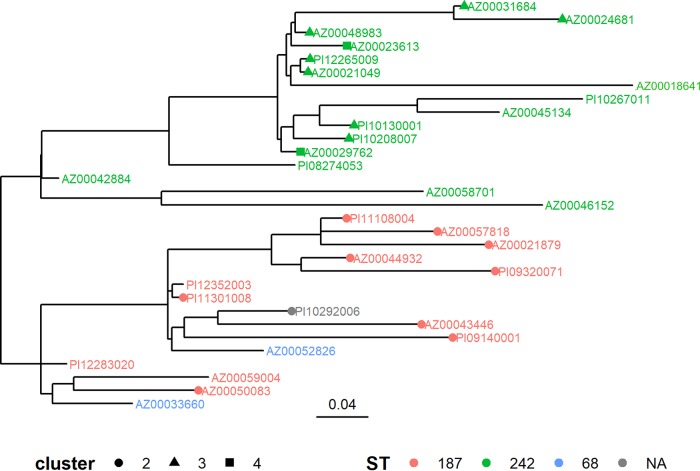
Pangenome analysis of clade A isolates. The presence or absence of accessory genes associated with the isolates in clade A from the wgMLST analysis was used to build the tree shown. Isolates are color coded by ST, and isolates associated with specific clusters as determined in the wgMLST analysis are depicted by different shapes. NA indicates that an ST could not be determined from the sequence data.

The pangenome of this clade contained 4,089 genes, of which 2,326 were core (i.e., genes present in ≥99% of the genomes). Similar to wgMLST, the pangenome analysis revealed that ST187 isolates could be distinguished from ST242 isolates; however, ST68 isolates did not cluster separately form ST187 isolates (Fig. 4). Moreover, isolates associated with clusters 2 to 4 did not form unique subclades in the pangenome analysis, suggesting that additional gene content was able to further differentiate many of these isolates compared to the wgMLST scheme used in this study.

## DISCUSSION

The incidence of legionellosis in Arizona is lower than the overall U.S. incidence and nearly 4-fold less than that reported for the states with the highest incidence of the disease (4). Compared to that for isolate submissions to CDC from other states, the proportion of Lp1 isolates from Arizona is smaller while the proportion of other serogroups (in particular, Lp6) is larger. Isolate submissions to the CDC from various states are not uniform, and detection of regional differences in serogroup distribution requires further study. Sources of isolate submission bias could include differences in public health laboratory capacity for *Legionella* culture and characterization, submission of only outbreak-related isolates, or submission of specific subsets such as non-Lp1 isolates.

Non-Lp1 strains have been detected in Arizona previously, including an outbreak involving an Lp6 strain in the potable water system of a hospital (9), a pseudo-outbreak associated with patients undergoing bronchoscopy where an Lp8 strain contaminated syringes during a bronchoscopy procedure that used ice made with the facility’s potable water (10), and more recently, both Lp1 and Lp6 were associated with legionellosis in neonates delivered in water births in tubs filled with tap water at private residences (11).

A total of 113 archived L. pneumophila isolates recovered from clinical specimens not known to be associated with outbreak investigations were selected for genome sequencing. Sequence-based typing (SBT) allele profiles were extracted from these genome sequences, and an ST was predicted for 78% of the genomes. wgMLST analysis revealed extensive diversity among genome sequences. One clade (clade A) was composed primarily of Lp6 isolates belonging to ST68, ST187, and ST242. Notably, the allele profile (*flaA*-*pilE*-*asd*-*mip*-*mompS*-*proA*-*neuA*) of ST68 (3**-** 13**-**1-28-14-9-3) differs by a single locus (*pilE*) with ST187 (3-10-1-28-14-9-3) and by two loci (*pilE* and *mompS*) with ST242 (3-10-1-28-1-9-3). Not surprisingly, ST242 isolates clustered separately from the other isolates using both wgMLST and pangenome analysis. Further analysis is required to determine if additional genome content associated with clade A could be utilized in the wgMLST scheme to improve the resolution of such sequences.

Previously, the utility of wgMLST was demonstrated for comparison of outbreak-related isolates recovered from clinical specimens and suspected environmental sources in New York State (8). In the current study, isolates were presumably associated with sporadic disease, yet 17 clusters of isolates sharing at least 99% allele identity were identified. This finding suggests that several strains of L. pneumophila capable of causing disease in humans may circulate in Arizona. It is also possible that this analysis detected previously unrecognized clusters of legionellosis. However, the detection of isolate clusters based on genome sequence analysis must be interpreted in the proper epidemiological context. Genome sequence analysis can further support the epidemiological relationships among isolates recovered from individuals or environmental sources but should not independently be used to infer that strains are associated with a particular source, as the population of strains causing sporadic disease are poorly understood.

The high diversity among isolates examined in this study supports the use of genome sequencing for routine analysis of isolates recovered from clinical sources. In this study, five isolates were recovered from individuals who resided out of state but whose clinical specimens were obtained in Arizona. wgMLST revealed that two of these isolates (AZ00025702 and PI11263001) shared <50% identical allele content with any of the other isolates examined. While it is unclear if these cases were travel associated, it is possible that a national genome sequence database could be used to compare sequences of clinical isolates recovered from individuals returning from travel with strains circulating in different regions. On a more local level, it was not possible to associate particular sequences or clades with various counties in Arizona, likely because the majority of isolates available were recovered from individuals residing in Maricopa County.

Exposure to a health care setting can be associated with some LD cases. In this study, two clusters contained isolates where some or all of the individuals had epidemiological links to a specific hospital over a period of multiple years. SNP analysis of the four isolates comprising cluster 8 revealed that three isolates recovered from individuals with exposure at a single facility (hospital B) were more closely related to each other than to the isolate recovered from an individual whose clinical specimen was obtained at a separate facility. Isolates associated with cluster 10 were recovered from five individuals residing in 4 separate counties and whose clinical specimens were obtained in four separate facilities. Two of these isolates sharing a single node were recovered from individuals treated at hospital C; however, these individuals also resided in counties in the southern part of Arizona while the remaining isolates were recovered from individuals residing in counties in the central part of the state.

In this study, serogroup 6 isolates appear to be more commonly isolated in Arizona than in other U.S. jurisdictions that share isolates with the CDC for serotype confirmation. Although comparative analysis of isolates from other jurisdictions may be affected by differences in isolate submission practices, the high rate of culture confirmation of legionellosis cases in Arizona reveals a highly diverse population of L. pneumophila strains capable of causing disease. While it remains to be rigorously tested, increased legionellosis incidence in this region due to L. pneumophila serogroup 6 would likely result in fewer cases detected using the urinary antigen test which primarily detects serogroup 1. The availability of clinical isolates has allowed analysis at the genomic level, demonstrating that the majority of serogroup 6 isolates recovered from clinical specimens in Arizona encompass more limited genetic diversity than serogroup 1 isolates. Additional analysis is required to determine if this difference in genomic diversity is restricted to isolates from Arizona or found in other regions as well. Interestingly, pangenome analysis suggested that, in some cases, additional gene content (compared to wgMLST loci) present among Lp6 isolates examined in this study may be useful for further strain resolution. Nevertheless, this work demonstrates that wgMLST is an effective tool for clustering genetically related isolates but must be interpreted in the context of epidemiological information. Finally, this work underscores the importance of sequencing L. pneumophila isolates in order to further understand the regional ecology and distribution of this organism.

## MATERIALS AND METHODS

### *Legionella* culture and isolate characterization.

*Legionella* species isolated from clinical specimens at various hospitals and clinical laboratories in Arizona were sent to the Arizona State Public Health Laboratory. Isolates were retained at −80°C for further study. In addition, isolates were sent to the CDC for confirmatory testing.

At the CDC, isolates were grown on BCYE and genomic DNA was extracted using the Epicentre MasterPure DNA isolation kit. A multiplex real-time PCR assay was used to detect markers for any *Legionella* spp., L. pneumophila, and Lp1 (12). Slide agglutination and direct fluorescence antibody testing were performed to determine the serogroup of L. pneumophila isolates that were not Lp1 (13, 14). Species determination for isolates that were not L. pneumophila was conducted using *mip* gene sequencing (15).

### Genome sequencing.

A subset of clinical L. pneumophila isolates collected between 2008 and 2017 present in the Arizona State Public Health Laboratory culture collection were sequenced in this study (see Table S1 in the supplemental material). Libraries were prepared using the Illumina Nextera XT kit and sequenced using 2 × 250 bp version 2 reagent kits with the Illumina MiSeq instrument.

### Bioinformatics analysis.

Raw sequence reads were analyzed using an L. pneumophila whole-genome multilocus sequence typing (wgMLST) method that is based on sequence variation present at a set of predefined loci (8). Briefly, a total of 5,778 loci were selected from representative L. pneumophila genomes, and sequence variations (alleles) were maintained in a centralized database to ensure reproducible subtyping results among various U.S. public health laboratories. Allele calling and comparative analysis of resulting wgMLST profiles were conducted using BioNumerics v. 7.5 (Applied Maths, Belgium). A combination of BLAST (assembly based) and a k-mer approach (assembly free) was used to detect alleles sharing at least 80% sequence similarity with reference loci (16). Separate alleles (containing a sequence variation) for a given locus were designated by a new allele number and catalogued using centralized allele database. A pairwise similarity matrix of allele profiles for each genome sequence was used to build an unweighted pair group method with arithmetic mean (UPGMA) dendrogram.

Genome assemblies were generated using Velvet (ver. 1.2.10) (17). In addition, seven genetic loci (*flaA*-*pilE*-*asd*-*mip*-*mompS*-*proA*-*neuA*) associated with a sequence-based typing (SBT) scheme were extracted from genomic sequence data (18). The allelic profile of these loci determines a sequence type (ST). Subsets of the genome alignments were used in a pangenome analysis which was conducted using Roary (v. 3.8.0) (19) and for comparison to the L. pneumophila strain Paris plasmid sequence (accession number NC_006365.1) using the BLAST Ring Image Generator (BRIG) v. 0.95 (20).

SNP analysis of ST1 isolates was conducted using CLC Genomic Workbench, v. 10 (Qiagen, Redwood City, CA, USA) for read mapping to the L. pneumophila ST1 strain Paris chromosome (accession number NC_006368.1). Consensus sequences were exported and aligned using MAFFT (v. 7.313) (21). The aligned sequences were analyzed using Gubbins to predict putative recombination regions (v. 2.3.1) (22). All phylogenetic trees were visualized using the R package ggtree (v. 1.10.5) (23).

### Data availability.

Raw sequence reads were deposited in the NCBI Sequence Read Archive (SRA) and are available under BioProject accession number PRJNA503807.
