# Supplementary figures and images for: Culture of Clinical Specimens Reveals Extensive Diversity of Legionella pneumophila Strains in Arizona
Source: mSphere. 2019 Feb 27;4(1):e00649-18. doi: 10.1128/mSphere.00649-18 (PMC6393729; doi:10.1128/mSphere.00649-18)

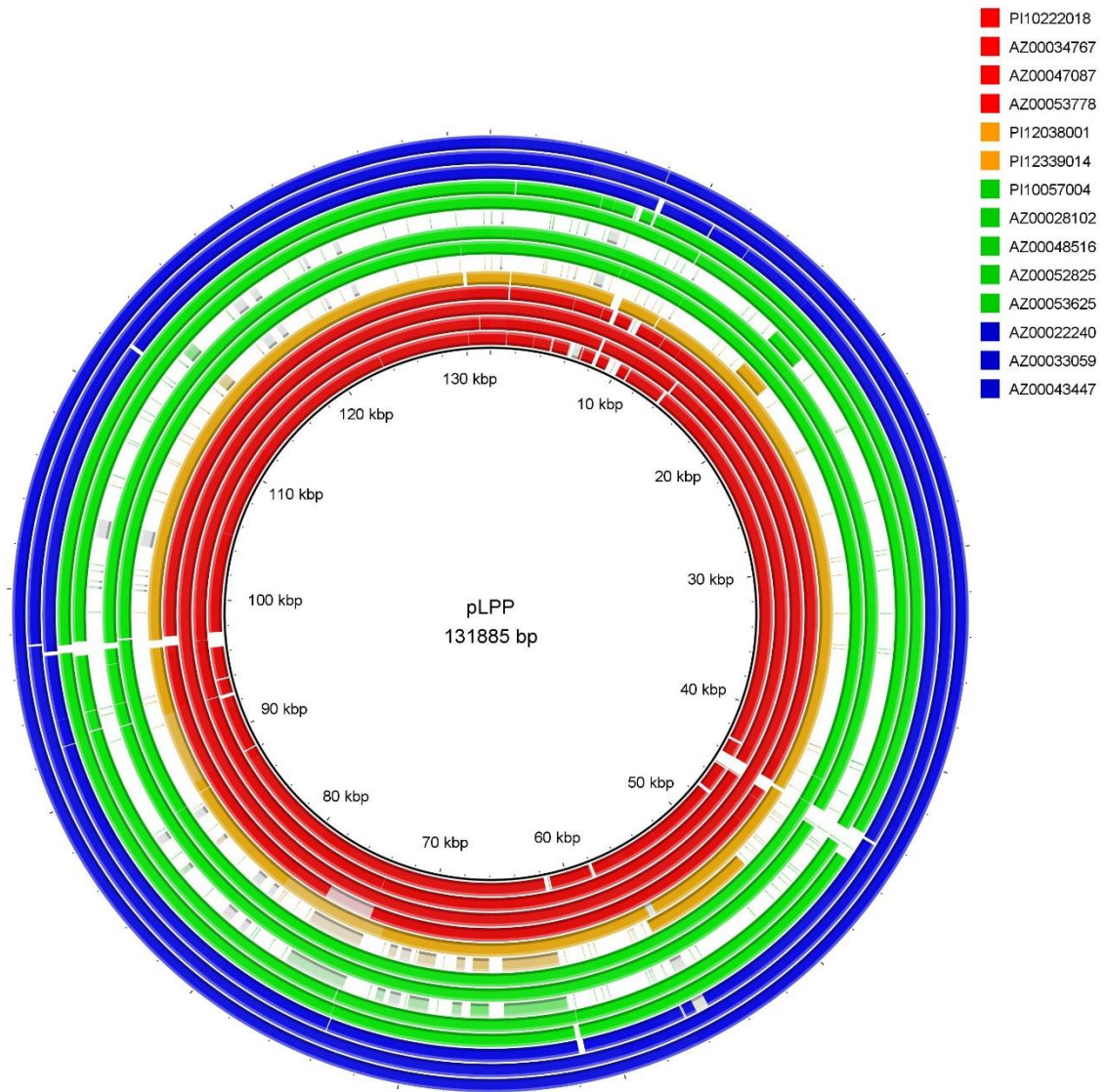

Supplement: FIG S1 [file mSphere.00649-18-sf001.pdf]
